# Editorial: Highlights in Thrombosis: 2021

**DOI:** 10.3389/fcvm.2022.863030

**Published:** 2022-02-24

**Authors:** Colin E. Evans

**Affiliations:** ^1^Program for Lung and Vascular Biology, Section for Injury, Repair and Regeneration, Stanley Manne Children's Research Institute, Ann & Robert H. Lurie Children's Hospital of Chicago, Chicago, IL, United States; ^2^Division of Critical Care, Department of Pediatrics, Northwestern University Feinberg School of Medicine, Chicago, IL, United States

**Keywords:** coagulation, research, thrombosis, 2021, vascular

In April 2021, the Thrombosis Section of *Frontiers in Cardiovascular Medicine* was launched. The aim of the Thrombosis Section is to publish high-quality, fundamental, translational, and clinical research on thrombotic diseases, as well as investigations of disorders driven by thrombo-inflammation. In this Editorial, studies from the Thrombosis Section of *Frontiers in Cardiovascular Medicine* in 2021 will be highlighted. Areas of opportunity for future studies and directions in thrombosis research will also be identified.

Venous thromboembolism (VTE) encompasses deep vein thrombosis (DVT) and pulmonary embolism (1, 2). Thrombosis and thromboembolic disease are leading causes of morbidity and mortality around the world (1, 2), with the incidence of VTE estimated to be 0.8 to 2.7 per 1,000 per year (3, 4). However, estimations of long-term survival after VTE are conflicting (5–15). In a prospective large cohort study, Nilius et al. aimed to assess long-term survival after VTE, as well as predictors of mortality. In this study, 6,243 patients with VTE from a University outpatient center in Switzerland were included; and records of clinical characteristics, disease severity, and treatments were analyzed. The standardized mortality ratio for the VTE patients vs. the general population of Switzerland was 1.3; in other words, long-term mortality of VTE patients was increased by 30%. The overall mortality rate of the VTE patients was 4.4 per 1,000 per year. Reduced survival was associated with unprovoked VTE, previous VTE, pulmonary embolism, permanent or prolonged anti-coagulant treatment, and cardiovascular co-morbidities. Even after adjustment for age, sex, and co-morbidities, the following variables remained as risk factors for mortality: unprovoked VTE, previous VTE, and permanent or prolonged anti-coagulation. In the previous conflicting studies referenced above, retrospective or case-control study designs with a small sample size were used, a limited number of variables for disease severity, treatment regimens, and co-morbidities were recorded, and/or the follow-up time was short. Strengths of the study by Nilius et al. include the large and prospective study cohort, the follow-up of patients for up to 30 years, and the analysis of a broad set of predictor variables. Methodological limitations were also discussed by Nilius et al. These included: (i) possible selection bias with high proportions of young and pregnant patients included: (ii) absence of data on the prescription of vitamin K antagonists or direct oral anti-coagulants; (iii) assessment of certain variables by self-reported questionnaire; and (iv) variable patient follow up times, with a minimum follow up time of ~1 year. Nevertheless, Nilius et al. provided a thorough analysis of long-term mortality after VTE and of predictors for survival in VTE patients. Given that long-term survival is reduced by VTE, future studies could improve survival by aiming to reduce thrombus formation, accelerate thrombus resolution, or limit the long-term complications of VTE (e.g., post-thrombotic syndrome). New therapeutic approaches could also be investigated, including precision medicine strategies for sub-populations of VTE patients with different disease severities and/or concurrent conditions.

Conditions that increase the prevalence of VTE include cancer, sepsis, and coronavirus disease 2019 (16–19). The incidence of VTE is increased to a greater extent in some cancer types vs. others (16, 20). In cancer patients, VTE is associated with increased morbidity and mortality (21, 22). It has also been suggested that the incidence of cancer-associated thrombosis (CAT) could differ from one geographic region to another, given circumstantial evidence that thrombogenicity could be dependent upon race (23, 24). Lee et al. performed a systematic review of CAT in Asia. These authors found that the estimated incidence of VTE in cancer patients from population-based studies in Asia is 1.9 to 9.9 per 1,000 per year. From hospital-based studies, Lee et al. found an even wider range of prevalence of VTE among cancer patients, from 5 to 446 per 1,000. The systematic review by Lee et al. therefore identified a need for large epidemiological studies of CAT in Asia, re-iterated the possibility that the incidence of CAT could vary by continent, and demonstrated that the type of study can affect estimates of CAT incidence. Factors that may alter such estimates include study design (e.g., prospective or retrospective; single center or multicenter), CAT diagnosis (e.g., incidental or clinically apparent VTE), patient cohort (e.g., inpatient or outpatient; surgical or medical), duration of study period and follow up, and cancer stage and type. Lee et al. concluded that the heterogeneity and variability of studies on CAT epidemiology in Asia demonstrate the importance of conducting carefully designed population-based studies in future. Other future directions identified by these authors include the generation of uniform patient registries to standardize data entry and analysis, and the generation and validation of standardized VTE risk scores in cancer patients.

Regarding other future directions for thrombosis research, a Specialty Grand Challenge article by Ten Cate identified 7 distinct but overlapping challenges. First, to reduce bleeding risk in patients receiving long-term anti-coagulant therapy. It was suggested that this could be achieved by selecting the optimal oral anti-coagulant, based on the clinical characteristics and biomarker profiles of the patient; and/or by developing novel anti-thrombotic agents with improved efficacy. Another challenge identified by Ten Cate is to tailor the anti-coagulant therapy to achieve a certain level of “dampening” of the coagulation cascade, i.e., without total inhibition. This approach would likely require an assessment of the patient's baseline coagulant activity. A third identified challenge in thrombosis research is to decipher the different cellular and molecular pathways that control different types of thrombosis. For instance, it could be interesting to determine whether the mechanisms that control thrombosis are consistent from one site to another. Another challenge is to better understand how thrombosis and the vascular response to thrombosis can impact upon other inflammatory conditions, including atherosclerosis and acute lung injury (25). Related to this, a fifth challenge identified was to optimize the use of laboratory research, with a view to improving clinical outcomes in VTE patients. While many pre-clinical tests and therapies fail in clinical trials, the article by Ten Cate provides several encouraging examples of laboratory research that aim to improve diagnostic testing for thrombosis and enhance the efficacy of anti-thrombotic agents. Another challenge identified by Ten Cate is to generate sufficient levels of funding to support cutting-edge research in the field of thrombosis. A final challenge is to leverage advanced technologies, including 3D vascular imaging and proteomics, genomics, and lipidomics, for the diagnosis and treatment of VTE.

New cell-specific biomarkers for thrombosis are currently being investigated, and novel 3D imaging techniques are being developed, to improve the diagnosis and treatment of thrombosis. In a recent review by Baidildinova et al., platelet-specific biomarkers for thrombosis and cardiovascular disease are described. Such biomarkers could be cellular proteins or secreted factors, the knowledge of which has increased rapidly in recent years. Baidildinova et al. concluded that the measurement of plasma biomarkers associated with VTE could facilitate the timely and personalized choice of therapy to prevent disease progression. In other words, novel biomarkers of platelet activation may prove valuable in the diagnosis and prognosis of VTE in future. Laboratory research should aim to identify biomarkers that are thrombosis-specific and can be measured rapidly and inexpensively in a timely manner. Novel histological imaging techniques that aim to provide 3D information on thrombus formation and resolution in lungs and other organs have been described elsewhere (19). In another example of 3D modeling, Liu et al. performed a retrospective evaluation of computed tomography scans and clinical data of 3 patients undergoing multi-branched endovascular repair. These authors reconstructed patient-specific 3D models and analyzed hemodynamic parameters for in-stent thrombosis (Liu et al.). Liu et al. concluded that hemodynamic perturbations in branched stent-grafts predispose to in-stent thrombosis, and that early hemodynamic analysis could help to identify in-stent thrombosis after multi-branched endovascular repair. Cellular and molecular assays are also being developed and assessed to improve the diagnosis and treatment of thrombosis. The thrombin generation assay is an example of an approach that has been used to measure the degree of anti-coagulation in VTE patients. Meihandoest et al. studied the correlation of thrombin generation with rivaroxaban, apixaban, and edoxaban drug dosages in a large, prospective, multi-center, cross-sectional study. A cohort of 559 patients were included in their study, but the correlation between thrombin generation and anti-coagulant drug concentration was weak, and drug levels were not correctly predicted by the thrombin generation assay. Future studies could aim to develop novel assays that can reliably assess the efficacy of anti-coagulant therapies in VTE patients.

As mentioned above, bleeding risk is an important consideration with anti-coagulant therapies (Ten Cate). The thrombin generation assay is an example of an approach that has also been investigated as a method of assessing bleeding risk in patients receiving dual anti-platelet therapy (de Breet et al.). In their study, coagulation factors and thrombin generation were measured in 93 high clinical-risk frail patients following percutaneous coronary intervention. Thrombin generation was measured at 1 and 6 months after percutaneous coronary intervention. At 12 months after percutaneous coronary intervention, clinically relevant bleeding events were assessed. At 1 month after percutaneous coronary intervention: thrombin generation, endogenous thrombin potential, peak height, and velocity index were lower in patients with bleeding compared to patients without bleeding. At 6 months after percutaneous coronary intervention: endogenous thrombin potential, peak height, and velocity index were decreased in the bleeding group compared with the non-bleeders. Authors concluded that thrombin generation assays have the potential to improve the identification of patients using dual anti-platelet therapy who are at risk of bleeding. A strength of the study by de Breet et al. is the use of the thrombin generation assays in patients, with prospective documentation of bleeding complications. However, the data is limited by the low sample size in certain groups and by the absence of follow-up data in some patients. Furthermore, baseline characteristics were different between patients with and without clinically relevant bleeding (i.e., differences in prevalence of anemia, transient ischemic attack/cerebrovascular accident, and malignancy).

Given that previous VTE is associated with increased mortality (Nilius et al.), another important consideration in thrombosis research is VTE recurrence. Nagler et al. developed and internally validated a prediction model for recurrent VTE in 479 patients with DVT. While their novel model showed some promise regarding the identification of predictors of VTE recurrence that have been confirmed by others (i.e., unprovoked DVT, male sex, increased D-dimer level, increased factor VIII expression, and inflammatory conditions), the study cohort was limited by several factors. As stated in their study, (i) the study included patients with proximal DVT only; (ii) only a very small number of cancer patients were included; (iii) factor VIII was measured after anti-coagulation; (iv) the number of patients with distal DVT as the recurrent event was not recorded; (v) age-adjusted D-dimer cut-offs were not included in the prediction model; and (vi) vitamin K-antagonists were being used in most patients. Newly developed models may therefore have the potential to predict bleeding risk in patients with DVT, but future studies should aim to verify such models in an independent setting of a larger cohort of DVT patients. Investigations of VTE recurrence in humans could be complemented in future studies by using experimental models of recurrent DVT (26).

Moving forward, preclinical and clinical studies of thrombosis should aim to contribute to the development of novel treatments that improve outcomes for patients with VTE. Experimental studies of thrombosis should also aim to improve the understanding and regulation of VTE. Great progress has been made in thrombosis research in recent years, but many research directions remain open.

## Author Contributions

The author confirms being the sole contributor of this work and has approved it for publication.

## Funding

CE is the recipient of an American Heart Association Career Development Award (19CDA34500000).

## Conflict of Interest

The author declares that the research was conducted in the absence of any commercial or financial relationships that could be construed as a potential conflict of interest.

## Publisher's Note

All claims expressed in this article are solely those of the authors and do not necessarily represent those of their affiliated organizations, or those of the publisher, the editors and the reviewers. Any product that may be evaluated in this article, or claim that may be made by its manufacturer, is not guaranteed or endorsed by the publisher.
